# Analysis of contingency tables based on generalised median polish with power transformations and non-additive models

**DOI:** 10.1186/2047-2501-1-11

**Published:** 2013-05-30

**Authors:** Frank Klawonn, Balasubramaniam Jayaram, Katja Crull, Akiko Kukita, Frank Pessler

**Affiliations:** Bioinformatics and Statistics, Helmholtz Centre for Infection Research, Inhoffenstr. 7, Braunschweig, D-38124 Germany; Ostfalia University of Applied Sciences, Salzdahlumer Str. 46/48, Wolfenbuettel, D-38302 Germany; Department of Mathematics, Indian Institute of Technology Hyderabad, Yeddumailaram, 502 205 India; Department of Molecular Immunology, Helmholtz Centre for Infection Research, Inhoffenstr. 7, Braunschweig, D-38124 Germany; Department of Microbiology, Saga Medical School, Saga, Japan; Department of Epidemiology, Helmholtz Centre for Infection Research, Inhoffenstr. 7, Braunschweig, D-38124 Germany

## Abstract

Contingency tables are a very common basis for the investigation of effects of different treatments or influences on a disease or the health state of patients. Many journals put a strong emphasis on p-values to support the validity of results. Therefore, even small contingency tables are analysed by techniques like t-test or ANOVA. Both these concepts are based on normality assumptions for the underlying data. For larger data sets, this assumption is not so critical, since the underlying statistics are based on sums of (independent) random variables which can be assumed to follow approximately a normal distribution, at least for a larger number of summands. But for smaller data sets, the normality assumption can often not be justified.

Robust methods like the Wilcoxon-Mann-Whitney-U test or the Kruskal-Wallis test do not lead to statistically significant p-values for small samples. Median polish is a robust alternative to analyse contingency tables providing much more insight than just a p-value.

Median polish is a technique that provides more information than just a p-value. It explains the contingency table in terms of an overall effect, row and columns effects and residuals. The underlying model for median polish is an additive model which is sometimes too restrictive. In this paper, we propose two related approach to generalise median polish. A power transformation can be applied to the values in the table, so that better results for median polish can be achieved. We propose a graphical method how to find a suitable power transformation. If the original data should be preserved, one can apply other transformations – based on so-called additive generators – that have an inverse transformation. In this way, median polish can be applied to the original data, but based on a non-additive model. The non-linearity of such a model can also be visualised to better understand the joint effects of rows and columns in a contingency table.

## Introduction

Contingency tables often arise from collecting patient data and from lab experiments. The rows and columns of a contingency table correspond to two different categorical attributes. One of these categorical attributes could account for different drugs with which patients are treated and the other attribute could stand for different forms of the same disease. Each cell of the table contains a numerical entry which reflects a measurement under the combination of the categorical attributes corresponding to the cell. In the example above, these entries could be the number of patients that have been cured from the disease by the drug corresponding to the cell. Or it could be the time or average time it took patients to recover from the disease while being treated with the drug.

Table 1 shows an example of a contingency table. The rows correspond to six different groups. The columns in this case reflect replicates. The columns correspond to 3 replicates of a gene expression experiment where cultured cells were transfected with increasing amounts of an effector plasmid (a plasmid expressing a protein that increases the expression of a gene contained on a second plasmid, referred to as a reporter plasmid) in the presence or absence of the reporter plasmid. Rows 1–3 constitute the negative control experiment, in which increasing amounts of the effector plasmid were transfected, but no reporter plasmid. The experiments in rows 4–6 are identical to those in 1–3, except that increasing amounts of the reporter plasmid were co-transfected. The data correspond to the intensity of the signal derived from the protein which is expressed by the reporter plasmid.

**Table 1:** **A contingency table**

Group		Replicate	
G1	6.39	8.10	6.08
G2	8.95	7.48	6.57
G3	5.61	8.58	5.72
G4	813.70	686.50	691.20
G5	4411.50	3778.90	4565.30
G6	32848.40	28866.00	46984.40

A typical question to be answered based on data from a contingency table is whether the rows or the columns show a significant difference. In the case of the treatment of patients with different drugs for different diseases, one could ask whether one of the drugs is more efficient than the other ones or whether one disease is more severe than the other ones. For the example of the contingency Table 1, one would be interested in significant differences among the groups, i.e. the rows. But it might also be of interest whether there might be significant differences in the replicates, i.e. the columns. If the latter question had a positive answer, this could be a hint to a batch effect, which turn out to be a serious problem in many experiments [1].

Hypothesis tests are a very common way to carry out such analysis. One could perform a pairwise comparison of the rows or the columns by the t-test. However, the underlying assumption for the t-test is that the data in the corresponding rows or columns originate from normal distributions. For very large contingency tables, this assumption is not very critical, since the underlying statistics will be approximately normal, even if the data do not follow a normal distribution. Non-parametric tests like the Wilcoxon-Mann-Whitney-U test are a possible alternative. However, for very small contingency tables they cannot provide significant p-values. In any case, a correction for multiple testing – like Bonferroni (see for instance [2]), Bonferroni-Holm [3] or false discovery rate (FDR) correction [4] – needs to be carried in the case of pairwise comparisons.

Instead of pairwise comparisons with correction for multiple testing, analysis of variance (ANOVA) is often applied instead of the t-test. Concerning the underlying model assumptions, ANOVA is even more restrictive than the t-test, since it does even assume that the underlying normal distributions have identical variance. ANOVA is also – like the t-test – very sensitive to outliers. The Kruskal-Wallis test is the corresponding counterpart of the Wilcoxon-Mann-Whitney-U test, carrying out a simultaneous comparison of the medians. But it suffers from the same problems as the Wilcoxon-Mann-Whitney-U test and is not able to provide significant p-values for small samples [5].

A general question is whether a p-value is required at all. A p-value can only be as good as the underlying statistical model and a lot of information is lost when the interestingness of a whole contingency table is just reflected by a single p-value. In the worst case, a t-test or ANOVA can yield a significant p-value just because of a single outlier.

Median polish [6] – a technique from robust statistics and exploratory data analysis – is another way to analyse contingency tables based on a simple additive model. We briefly review the idea of median polish in terms of a simple additive model. Although the simplicity of median polish as an additive model is appealing, it is sometimes too simple to analyse contingency table. Very often, especially in the context of gene, protein or metabolite expression profile experiments, the measurements are not taken directly, but are transformed before further analysis. In the case of expression profiles, it is common to apply a logarithmic transformation. The logarithmic transformation is a member of a more general family, the so-called power transformations which we use to introduce a method to find a suitable power transformation that yields the best results for median polish for a given contingency table. The leads to median polish based on an additive model, but with transformed attribiutes. We further extend the presented ideas, by transforming the median polish back to the original domain of the attributes. This back-transformation requires special transformations related to additive generators. With such back-transformation the median polish result can be interpreted on the original data values as non-additive model. Finally, we illustrate how to visualise the non-linearity exploited by the non-additive median polish model. This paper combines the ideas that were presented in [7, 8].

## Median polish

Median polish has been applied to medical and biomedical contingency tables in various settings [9–11]. The underlying additive model of median polish is that each entry *x*_*ij*_ in the contingency table can be written in the form

- *g* represents the overall or grand effect in the table. This can be interpreted as general value around which the data in the table are distributed.
- *r*_*i*_ is the row effect reflecting the influence of the corresponding row *i* on the values.
- *c*_*j*_ is the column effect reflecting the influence of the corresponding column *j* on the values.
- *ε*_*ij*_ is the residual or error in cell (*i*,*j*) that remains when the overall, the corresponding row and column effect are taken into account.

The overall, row and column effects and the residuals are computed by the following algorithm.

1. For each row compute the median, store it as the row median and subtract it from the values in the corresponding row.
2. The median of the row medians is then added to the overall effect and subtracted from the row medians.
3. For each column compute the median, store it as the column median and subtract it from the values in the corresponding column.
4. The median of the column medians is then added to the overall effect and subtracted from the column medians.
5. Repeat steps 1–4 until no changes (or very small changes) occur in the row and column medians.

Table 2 shows the result of median polish applied to Table 1.

**Table 2:** **Median polish for the data in Table** 1

		Overall: 350.075		
	R1	R2	R3	Row effect
G1	0.000	4.795	-0.310	-343.685
G2	0.000	1.615	-2.380	-341.125
G3	-0.110	5.945	0.000	-344.355
G4	122.500	-1.615	0.000	341.125
G5	0.000	-629.515	153.800	4061.425
G6	0.000	-3979.315	14136.000	32498.325
Column effect	0.000	-3.085	0.000	

The result of median polish can help to better understand the contingency table. In the ideal case, the residuals are zero or at least close to zero. Close to zero means in comparison to the row or column effects. If most of the residuals are close to zero, but only a few have a large absolute value, this is an indicator for outliers that might be of interest. Most of the residuals in Table 1 are small, except the ones in the lower right part of the table.

The row effect shows how much influence each row, i.e. in the example, each group has. One can see that group G1, G2 and G3 have roughly the same effect. Group G5 and G6 have extremely high influence and show very significant effects.

The column effects are interpreted in the same way. Since the columns represent replicates, they shall have no effect at all in the ideal case. Otherwise, some batch effect might be the cause. The column effects in Table 1 are – as expected – all zero or at least close to zero.

## Power transformations

Transformation of data is a very common step of data preprocessing (see for instance [12]). There can be various reasons for applying transformations before other analysis steps, like normalisation, making different attribute ranges comparable, achieving certain distribution properties of the data (symmetric, normal etc.) or gaining advantage for later steps of the analysis.

Power transformations (see for instance [6]) are a special class of parametric transformations defined by 

It is assumed that the data values *x* to be transformed are positive. If this is not the case, a corresponding constant ensuring this property should be added to the data.

We restrict our considerations on power transformations that preserve the ordering of the values and therefore exclude negative values for *λ*.

In the following section, we use power transformations to improve the results of median polish.

## Finding a suitable power transformations for median polish

An ideal result for median polish would be when all residuals are zero or at least small. The residuals get smaller automatically when the values in the contingency table are smaller. This would mean that we tend to put a high preference on the logarithmic transformation (*λ* = 0), at least when the values in the contingency table are greater than 1. Small for residuals does not refer to the absolute values of the residuals being small. It means that the residuals should be small compared to the row or column effects. Therefore, we should compare the absolute values of the residuals to the absolute values of the row or column effects. One way to do this would be to compare the mean values of the absolute values of the residuals to the mean value of the absolute values of the row or column effects. This would, however, be not consistent in the line of robust statistics. Single outliers could dominate this comparison. This would also lead to the reverse effect as considering the residuals alone. Power transformations with large values for *λ* would be preferred, since they make larger values even larger. And since the row or column effects tend to be larger than the residuals in general, one would simply need to choose a large value for *λ* to emphasize this effect.

Neither single outliers of the residuals nor of the row or column effects should have an influence on the choice of the transformation. What we are interested in is being able to distinguish between significant row or column effects and residuals. Therefore, the spread of the row or column effects should be large whereas at least most of the absolute values of the residuals should be small.

To measure the spread of the row or column effects, we use the interquartile range which is a robust measure of spread and not sensitive to outliers like the variance. The interquartile range is the difference between the 75%- and the 25%-quantile, i.e. the range that contains 50% percent of the data in the middle.

We use the 80% quantile of the absolute values of all residuals to judge whether most of the residuals are small. It should be noted that we do not expect all residuals to be small. We might have single outliers that are of high interest.

Finally, we compute the quotient of the interquartile range of the row or column effects and divide it by the 80% quantile of the absolute values of all residuals. We call this quotient the IQRoQ value (**I**nter**Q**uartile **R**ange **o**ver the 80% **Q**uantile of the absolute residuals). The higher the IQRoQ value, the better is the result of median polish. For each value of *λ*, we apply the corresponding power transformation to the contingency table and calculate the IQRoQ value. In this way, we obtain an IQRoQ plot, plotting the IQRoQ value depending on *λ*.

Of course, the choice of the interquartile range – we could also use the range that contains 60% percent of the data in the middle – and the 80%-quantile for the residuals are rules of thumb that yield good results in our applications. If more is known about the data, for instance that outliers should be extremely rare, one could also choose a higher quantile for the residuals.

Before we come to examples with real data in the next section, we illustrate our method based on artificially generated contingency tables. The first table is a 10 × 10, generated by the following additive model. The overall effect is 0, the row effects are 10,20,30,…,100, the column effects are 1,2,3,…,10. We then added to each entry noise from a uniform distribution over the interval [-0.5,0.5] to each entry.

**Figure 1:**
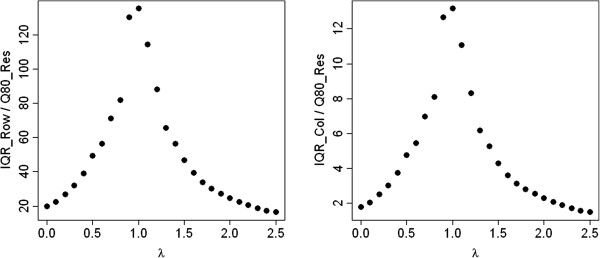
**IQRoQ plot for the row (left) and column effects (right) for the artificial example data set.**

Figure 1 shows the IQRoQ plots for the row and column effects for this artificial data set. In both cases, we have a clear maximum at *λ* = 1. So the IQRoQ plots propose to apply the power transformation with *λ* = 1 which is the identity transformation and leaves the contingency table as it is. The character of the IQRoQ plots for the row and column effects is similar, but the values differ by a factor 10. This is in complete accordance with the way the artificial data set had been generated. The row effects were chosen 10 times as large as the column effects.

**Figure 2:**
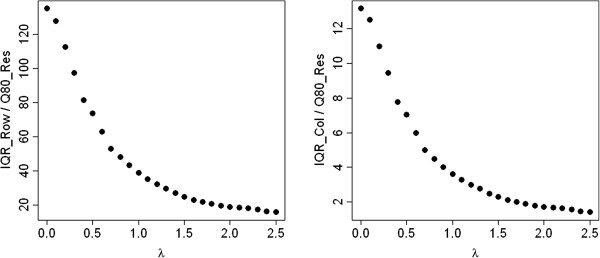
**IQRoQ plot for the row (left) and column effects (right) for the exponential artificial example data set.**

As a second artificial example we consider the same contingency table, but apply the exponential function to each of its entries. The IQRoQ plots shown in Figure 2 have their maximum at *λ* = 0 and therefore suggest to use the logarithmic transformation before applying median polish. So this power transformation reverses the exponential function and we retrieve the original data which were generated by the additive model.

**Figure 3:**
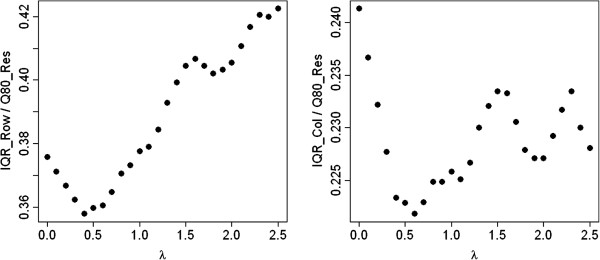
**IQRoQ plot for the row (left) and column effects (right) for a random data set where all entries in the contingency table were generated by a normal distribution with expected value 5 and variance 1.**

The last artificial example is a negative example in the sense that there is no additive model underlying the data generating process. The entries in the corresponding 10 × 10 contingency table were produced by a normal distribution with expected value 5 and variance 1. The IQRoQ plots are shown in Figure 3. The IQRoQ plot for the row effect has no clear maximum at all and shows a tendency to increase with increasing *λ*. The IQRoQ plot for the column effect has a maximum at 0 and then seems to oscillate with definitely more than one local maximum. There is no clear winner among the power transformations. And this due to the fact that there is no underlying additive model for the data and no power transformation will make the data fit to an additive model.

## Examples

**Figure 4:**
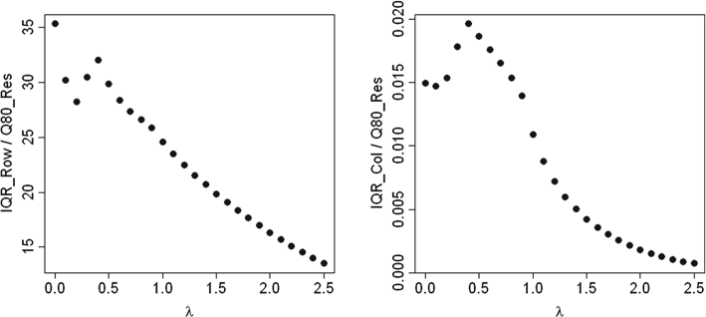
**IQRoQ plot for the row (left) and column effects (right) for the data set in Table** 1.

We now apply the IQRoQ plots to real data sets. As a first example, we consider the data set in Table 1. The IQRoQ plots are shown in Figure 4. The IQRoQ plot for the row effects has its global maximum at *λ* = 0 and a local maximum at *λ* = 0.5. The IQRoQ plot for the column effects has its global maximum at *λ* = 0.5. However, we know that in this data set the columns correspond to replicates and it does not make sense to maximise the effects of the replicates over the residuals. The IQRoQ values for the column effects are also much smaller than the IQRoQ values for the row effects. Therefore, we chose the power transformation suggested by the IQRoQ plot for the row effects, i.e. the logarithmic transformation induced by *λ* = 0. The second choice would be the power transformation with *λ* = 0.5 which would lead to similar effects as the logarithmic transformation, although not so strong.

Table 3 shows the result of median polish after the logarithmic transformation has been applied to the data in Table 1. We compare this table with Table 2 which originated from median polish applied to the original data. In Table 3 based on the optimal transformation derived from the IQRoQ plots, the absolute values of all residuals are smaller than any of the (absolute) row effects. There is no indication of extreme outliers anymore, whereas the median polish in Table 2 applied to the original data suggests that there are some extreme outliers. The entries for G6 for replicate R2 and R3 and even the entry for G5 for replicate R2 show a larger absolute value of the majority of the row effects in Table 2. From Table 2, it is also not very clear whether group G4 is similar to groups G1, G2, G3 or groups G5, G6, whereas after the transformation in Table 1 the original groupings G1, G2, G3 (no reporter plasmid) versus of G4, G5, G6 (with increasing amount of reporter plasmid) can be easily identified based on the row effects.

**Table 3:** **Median polish for the data in Table** 1 after power transformation with ***λ*** **=** **0**

		Overall: 4.2770		
	R1	R2	R3	Row effect
G1	0.0000	0.2422	-0.0497	-2.4223
G2	0.1760	0.0017	-0.1331	-2.2614
G3	-0.0194	0.4106	0.0000	-2.5331
G4	0.1632	-0.0017	0.0000	2.2614
G5	0.0000	-0.1497	0.0343	4.1149
G6	0.0000	-0.1241	0.3579	6.1226
Column effect	0.0000	-0.0051	0.0000	

We finally consider two larger contingency tables with 14 rows and 97 columns that are far too large to be included in this paper. The tables consist of a data set displaying the metabolic profile of a bacterial strain after isolation from different tissues of a mouse. The columns reflect the various substrates whereas the rows consist of repetitions for the isolates from tumor and spleen tissue. The aim of the analysis is to identify those substrates that can be utilized by active enzymes and to find differences in the metabolic profile after growth in different organs.

**Figure 5:**
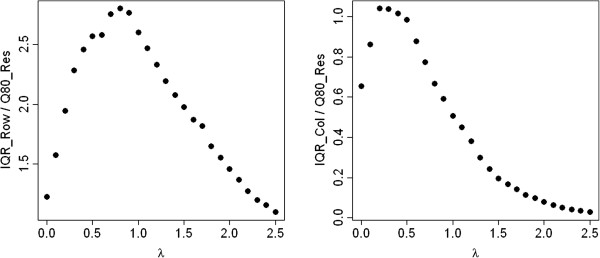
**IQRoQ plot for the row (left) and column effects (right) for a larger contingency table for spleen.**

**Figure 6:**
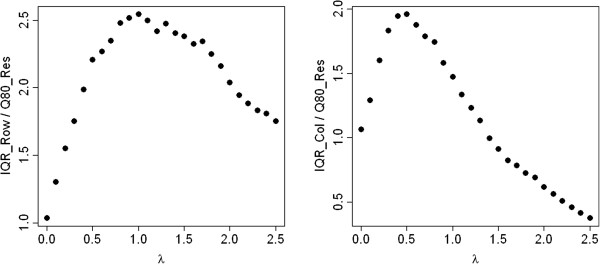
**IQRoQ plot for the row (left) and column effects (right) for a larger contingency table for tumour.**

The corresponding IQRoQ plots are shown in Figures 5 and 6. The IQRoQ plots indicate that we choose a value of around *λ* = 0.5, although the IQRoQ plots do not agree on exactly the same value.

## The non-additive model

In the previous setting, we have looked at the median polish results for the transformed data. Sometimes, transformations of the attributes might not be desired, since the transformed attribute might not be interpretable for the domain expert anymore. Therefore, we introduce transformations that can be reversed leading to median polish on the original attributes based on non-additive models. In order to motivate and explain this idea, we take a closer look at the power transformation with *λ* = 0, i.e. we when choose the logarithm for the power transformation. We then obtain the following model. 1

Transforming back to the original data yields the model 

So it is in principle a multiplicative model (instead of an additive model as in standard median polish) as follows: 

where , , , . The part of the model which is not so nice is that the residuals also enter the equation by multiplication. Normally, residuals are always additive, no matter what the underlying model for the approximation of the data is.

Towards overcoming this drawback, we propose the following approach. We apply the median polish algorithm to the log-transformed data in order to compute *g* (or ), *r*_*i*_ (or ) and *c*_*j*_ (or ). The residuals are then defined at the very end as 2

Let us now rewrite Eq. (1) in the following form: 

Assuming that the residuals are small, we have 

Transforming this back to the original data, we obtain 

A natural question that arises now is the following: *What happens with other power transformations, i.e., forλ* > 0? In principle the same, as we obtain 3

Let us denote by ⊕_*λ*_ the corresponding, possibly associative, operator obtained as follows: 4

Now, we can interpret Eq. (3) as 5

Thus the problem of determining a suitable transformation of the data before applying the median polish algorithm essentialy boils down to finding that operator ⊕_*λ*_ which minimises the residuals in (2), viz., 

## Transformations and additive generators of fuzzy logic connectives

It is very interesting to note the similarity between the operator ⊕_*λ*_ and t-norms / t-conorms [13], operators for modelling the AND, respectively the OR operator in fuzzy logic.

On the one hand, the above family of power transformations closely resembles the Schweizer-Sklar family of additive generators^a^ of t-norms. In fact, the power transformations are nothing but the negative of the additive generator of the Schweizer-Sklar t-norms. Note that additive generators of t-norms are non-increasing, and in the case of continuous t-norms they are strictly decreasing, which explains the need for a negative sign to make the function decreasing.

On the other hand, given continuous and strict additive generators, one constructs t-norms / t-conorms precisely by using Eq. (4).

However, it should be emphasised that additive generators of t-norms or t-conorms cannot be directly used here. The additive generator of a t-norm is non-increasing while one requires a transformation to maintain the monotonicity in the arguments. In the case of the additive generator of a t-conorm, though monotonicity can be ensured, their domain is restricted to just [0,1]. This can be partially overcome by normalising the data to fall in this range. However, this type of normalisation may not be reasonable always. Further, the median polish algorithm applied to the transformed data do not always remain positive and hence determining the inverse with the original generator is not possible.

The above discussion leads us to consider a suitable modification of the additive generators of t-norms / t-conorms that can accommodate a far larger range of values both in their domain and co-domain. Representable uninorms are another class of fuzzy logic connectives that are obtained by the additive generators of both a t-norm and a t-conorm. In this work, we construct newer transformations by suitably modifying the underlying generators of these representable uninorms [13].

### Modified additive generators of uninorms : an example

Let us assume that the data *x* are coming from the interval (-*M*,*M*). Consider the following modified generator of the uninorm obtained from the additive generators of the Schweizer-Sklar family of t-norms and t-conorms.

Let *e*∈(-*M*,*M*) be any arbitrary value. Then the following is a valid transformation with , for all *λ* ≠ 0. 

Note that *h*_*λ*_ is monotonic for all *λ* ≠ 0 and increases with decreasing *λ*.

**Figure 7:**
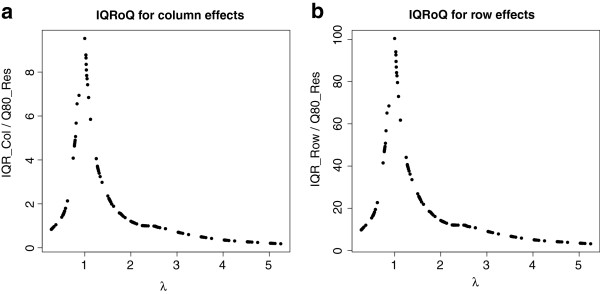
**IQRoQ plots for the column and row effects of the artificial data with Modified Schweizer-Sklar generator.** (**a**) Artificial Data, *e* = 5,*L* = 110, IQRoQ Column Plot. (**b**) Artificial data, *e* = 5,*L* = 110, IQRoQ Row Plot.

That this modified generator is a reasonable transformation can be seen by applying it to the random data set that was already used to generate the IQRoQ plots in Figure 1. From the IQRoQ plots for this data given in Figure 7, it can be seen that the global maxima occur at *λ* = 1. So the IQRoQ plots propose to apply the above transformation with *λ* = 1 which is a linear transformation of the data.

### A novel way of finding a suitable transformation

In this section we present the algorithm to find a suitable transformation of the given data such that the MP algorithm performs well to elucidate the underlying structures in the data. We only consider a one parameter family of operators with the parameter denoted by *λ*.

The proposed algorithm is as follows. Let ⊕_*λ*_ denote the one parameter family of operators whose domain and range allow it to be operated on the data given in the contingency table. Then for each *λ* the following steps are performed:

1. Apply the transformation ⊕_*λ*_ to the contingency table.
2. Apply median polish to the transformed data to find the overall, row and column effects, viz.,  for each *i*,*j*.
3. Find the residuals  for each *i*,*j*.
4. Determine the IQRoQ values of the above residuals.

Finally, we plot *λ* versus the above IQRoQ values to get the IQRoQ plots for the column and row effects.

Clearly, the operator corresponding to the *λ* at which the above IQRoQ plots peak is a plausible transformation for the given contingency table.

### Some illustrative examples

**Figure 8:**
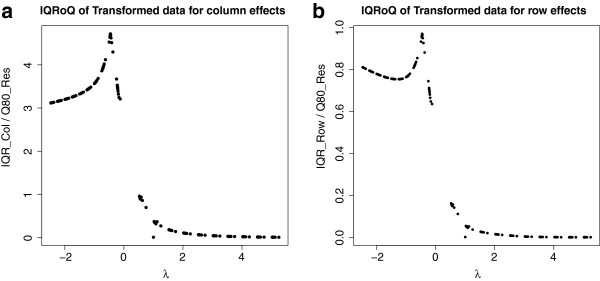
**IQRoQ plots for the column and row effects of the Infant Mortality data.** (**a**) *e* = 2,*M* = 40, IQRoQ Column Plot. (**b**) *e* = 2,*M* = 40, IQRoQ Row Plot.

As an example with real world data, let us consider the data given in the contingency Table 4. Applying the above algorithm with the transformation *h*_*λ*_ we obtain the following IQRoQ plots as detailed above. The corresponding IQRoQ plots are shown in Figures 8(a) and (b). The IQRoQ plots suggest a value of around *λ* = -0.5. The ‘median polished’ contingency table for *λ* = -0.5 is given in Table 5.

**Table 4:** **Infant mortality vs educational qualification of the parents in deaths per 1000 live births in the years 1964–1966 (Source: U.S. Dept. of Health, Education and Welfare)**

	***≤8***	9–11	***12***	13–15	***≥16***
North-West	25.3	25.3	18.2	18.3	16.3
North-Central	32.1	29.0	18.8	24.3	19.0
South	38.8	31.0	19.3	15.7	16.8
West	25.4	21.1	20.3	24.0	17.5

**Table 5:** **Median polish on the** ***h***_**λ**_ **- transformed infant mortality data with** ***λ*** **=** **-** **0** **.** **5**

			Overall: 0.2919985			
	***≤8***	9–11	12	13–15	***≥16***	***RE***
NW	0.00025312	0.0027983	-0.00025004	-0.010879	0.0000000	-0.010113225
NC	-0.00025312	-0.0027983	-0.01200293	0.010879	0.0078014	0.006694490
S	0.01098492	0.0091121	0.00025004	-0.044525	-0.0035433	-0.001558958
W	-0.01102793	-0.0305895	0.00456985	0.014641	0.0000000	0.001558958
*CE*	0.0318984143	0.0293532152	-0.0112376220	0.0002531186	-0.0294192135	

**Figure 9:**
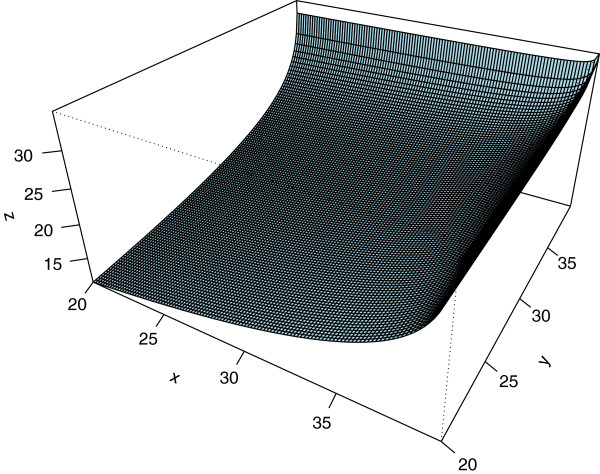
**The operator for the non-additive median polish model for the Infant Mortality data.**

We can also visualise the non-linear aggregation operator ⊕_*λ*_ (here: *λ* = -0.5) that is used for the non-additive median polish model. The non-linearity is clearly illustrated in Figure 9 which suggests that strong row and column effects seem to even amplify each other.

**Figure 10:**
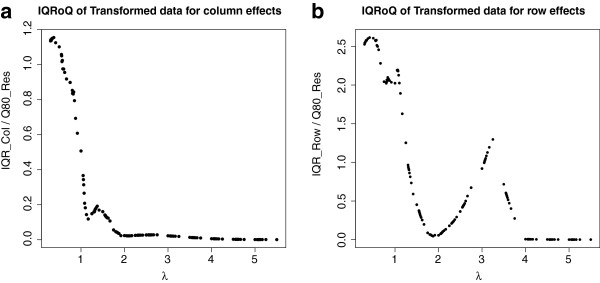
**IQRoQ plots for the column and row effects of the Spleen data.** (**a**) *e* = 10,*M* = 20000, IQRoQ Column Plot. (**b**) *e* = 10,*M* = 20000, IQRoQ Row Plot.

We also apply the non-additive median polish model to the data set that was already used for Figure 5. The corresponding IQRoQ plots are shown in Figures 10(a) and (b). The IQRoQ plots indicate that we choose a value of around *λ* = 0.4.

### An example based on clinical data

We consider a data set from [14] containing a sample of male residents of Framingham in Massachusetts shown in Table 6.

**Table 6:** **Median polish for the data in Table** 7

				Overall: 3.625				
	C1	C2	C3	C4	C5	C6	C7	Row effect
P1 absent	33.000	5.000	-1.500	-0.125	-2.625	3.625	-5.625	13.125
P2 absent	40.500	1.500	0.000	33.375	-0.125	-7.875	-6.125	20.625
P3 absent	22.750	1.750	-13.750	44.625	0.125	-9.625	-8.875	29.375
P4 absent	36.500	-6.500	0.000	45.375	-6.125	5.125	-7.125	22.625
P5 absent	17.000	0.000	-10.500	5.875	0.375	-3.375	-1.625	13.125
P6 absent	0.000	0.000	0.5000	-0.125	0.375	-0.375	-2.625	7.125
P7 absent	8.750	-5.250	0.250	10.625	-7.875	-1.625	0.125	7.375
P8 absent	-2.750	-3.750	-2.250	0.125	0.625	-0.125	0.625	1.875
P1 present	0.500	0.500	0.000	-3.625	-0.125	0.125	-0.125	-3.375
P2 present	-2.500	1.500	0.000	3.375	-1.125	3.125	-0.125	-2.375
P3 present	0.000	0.000	1.500	-2.125	-0.625	0.625	3.375	-2.875
P4 present	-1.750	-0.750	-1.250	1.125	1.625	-0.125	2.625	-2.125
P5 present	0.000	0.000	-0.500	-1.125	1.375	2.625	0.375	-2.875
P6 present	-0.500	0.500	0.000	-2.625	-0.125	1.125	3.875	-3.375
P7 present	0.000	-1.000	-1.500	-3.125	0.375	3.625	1.375	-1.875
P8 present	-1.000	0.000	2.500	-3.125	0.375	-0.375	0.375	-2.875
Column effect	1.250	-0.750	-0.250	3.375	-0.125	0.625	-0.125	

The age of the persons ranges between 40 and 59 year. Several attributes were taken into accout, among them blood pressure and cholesterol level. The persons were classified whether they developed a coronary heart disease within a period of six years. The blood pressure was divided into eight levels, P1 referring to the lowest level (<117), P2 to a blood pressure between 117 and 126 etc. and P8 corresponding to blood pressures above 186. Similar to the blood pressure, the cholesterol level was divided into seven groups (C1: <200, C2: 200–209, C3: 210–219, C4: 220–244, C5: 245–259, C6: 260–284, C7: >284).

As one would expect from such a study, the number of observed cases with coronary disease within this six year period is relatively small compared to number of persons not being classified as having a coronary disease. This makes it quite difficult to see what would be expected, namely that a high level of cholesterol and high blood pressure increase the risk of coronary disease.

Table 7 shows the result of applying median polish without any transformation to Table 6. This table contains large residuals, the largest absolute residual of 45.375 at (P4 absent,C3) exceeds by far the row and column effects. The absolute values of the residuals also exhibit a large variation. The relative variance of the absolute residuals is 18.192. The principal expected effects can be guessed from the median polish result, but could be doubted due to the large residuals compared to the row and column effects. It is obvious to expect a positive row effect for the first eight rows, i.e. for the persons who did not show any signs of heart disease, simply because this group of persons forms the large majority in the table. We would also expect that this positive effect is smaller for larger levels of the blood pressure. This can be observed, but these effects do not look significant compared to the large residuals. The column effects, i.e. the cholesterol levels, seem to have a small influence. None of the column effects is larger than the mean (4.504) of the absolute residuals, all column effects are even smaller than the median (1.438) of the absolute residuals.

**Table 7:** **Coronary disease data from [**[14]**]**

					Cholesterol level			
Heart disease	Pressure	C1	C2	C3	C4	C5	C6	C7
Absent	P1	51	21	15	20	14	21	11
Absent	P2	66	25	24	61	24	17	18
Absent	P3	57	34	19	81	33	24	24
Absent	P4	64	19	26	75	20	32	19
Absent	P5	35	16	6	26	17	14	15
Absent	P6	12	10	11	14	11	11	8
Absent	P7	21	5	11	25	3	10	11
Absent	P8	4	1	3	9	6	6	6
Present	P1	2	0	0	0	0	1	0
Present	P2	0	2	1	8	0	5	1
Present	P3	2	0	2	2	0	2	4
Present	P4	1	0	0	6	3	2	4
Present	P5	2	0	0	3	2	4	1
Present	P6	1	0	0	1	0	2	4
Present	P7	3	0	0	2	2	6	3
Present	P8	1	0	3	1	1	1	1

**Figure 11:**
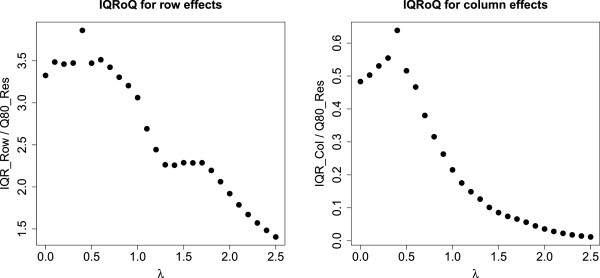
**IQRoQ plot for the row (left) and column effects (right) for the data in Table** 7.

Since we have zero values in the table, we cannot apply the logarithmic power transformation to the data. In order to avoid this problem, we apply Laplace correction, i.e. we add a positive constant, say 1, to all entries in the table. The IQRoQ plots for the Laplace corrected data set, shown in Figure 11, indicate that a value for *λ* around 0.4 yields the most suitable power transformation.

Table 8 shows the result of median polish applied to the transformed data. The residuals are now smaller compared to the row and column effects. The largest absolute residual is 3.756 at (P1 absent,C1). Even this largest residual is smaller than three of the row effects which can then be considered significant. Also the relative variance of the absolute values of the residuals is much smaller now. It is only 0.897. Now there is also one column effect which is larger than the mean (0.787) of the absolute residuals and two column effects are larger than the median (0.611) of the absolute residuals.

**Table 8:** **Median polish for the data in Table** 7 **after power transformation with** ***λ*** **=** **0** **.** **4**

				Overall: 1.756				
	C1	C2	C3	C4	C5	C6	C7	Row effect
P1 absent	3.756	1.130	0.000	-0.520	-0.243	0.301	-0.849	3.423
P2 absent	3.570	0.244	0.000	2.578	-0.050	-1.844	-0.967	4.904
P3 absent	1.731	0.320	-1.859	3.034	0.050	-1.815	-1.111	5.990
P4 absent	3.125	-0.956	0.000	3.401	-0.943	0.052	-1.082	5.187
P5 absent	1.829	0.021	-2.400	0.108	0.050	-1.188	-0.291	3.689
P6 absent	0.000	0.459	0.589	-0.170	0.539	-0.139	-0.170	2.010
P7 absent	1.094	-1.485	0.050	1.107	-2.402	-0.910	0.025	2.549
P8 absent	-0.818	-1.368	-0.415	0.121	0.627	-0.052	0.652	0.612
P1 present	0.570	0.101	0.000	-1.390	-0.050	0.070	-0.025	-1.656
P2 present	-1.608	0.682	0.000	1.331	-0.849	1.092	-0.025	-0.857
P3 present	0.000	-0.470	0.809	-0.581	-0.620	0.080	1.664	-1.085
P4 present	-0.713	-0.602	-0.703	0.851	1.100	-0.052	1.531	-0.953
P5 present	0.000	-0.470	-0.570	-0.108	0.759	0.960	0.203	-1.085
P6 present	-0.010	0.101	0.000	-0.592	-0.050	0.651	2.234	-1.656
P7 present	0.000	-0.943	-1.044	-1.054	0.286	1.172	0.784	-0.612
P8 present	-0.132	-0.021	1.731	-0.714	0.627	-0.052	0.652	-1.534
Column effect	0.709	-0.201	-0.100	1.291	-0.050	0.629	-0.075	

**Figure 12:**
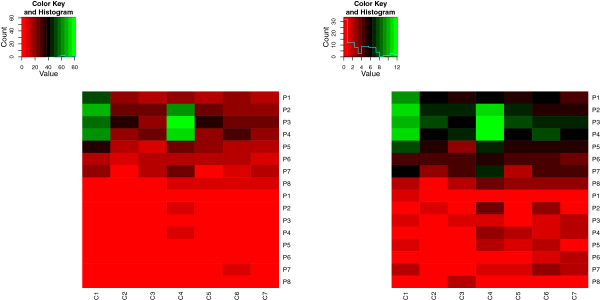
**Heatmap visualisation of the data from Table** 7 **(left) and the data after transformation (right).**

It is also interesting to take a look at the transformed data set that was found based on the IQRoQ plots. Figure 12 visualises the original (left) and the transformed (right) contingency table. Both table show a tendency of higher values in the upper half (persons with absent heart disease). But the difference between the upper and the lower half is much clearer for the the transformed contingency table than for the original one. This means that even without applying median polish, it might be useful to look at the transformed contingency table generated by the transformation derived from the IQRoQ plots.

## Conclusions

We have proposed two methods to improve the results of median. Either we apply a suitable power transformation to the data before applying median polish. Based on the IQRoQ plots, the most suitable power transformation can be chosen. Or, as an alternative, one can apply reversible transformations based on additive generators, leading to non-additive median polish. Again, the most suitable reversible transformation is chosen based on IQRoQ plots. The joint non-linear connection of column and row effects can be visualsied by a function in two variables in order to better understand the nature of the interaction of column and row effects. The example on heart disease has demonstrated that it can be useful to apply a transformation derived from IQRoQ plots, even if it is not necessarily intended to use median polish afterwards. The transformed contingency table might already exhibit a clearer structure than the original table.

## Ethical approval

All data sets referred to in this manuscript have been published before and were in compliance with the Helsinki Declaration. No specific or additional experiments were carried out for this manuscript.

## Software

The IQRoQ plots in this paper were generated by an implementation of the described method in R, a free software environment for statistical computing and graphics [15] (see http://www.r-project.org/). The simple R implementation for generating IQRoQ plots can be downloaded at http://public.ostfalia.de/~klawonn/hiss_mp.R.

## Endnote

^**a**^ An additive generator of a function *f*(*x*,*y*) in two real variables is a function *h* in one real variable such that *f*(*x*,*y*) = *h*^-1^(*h*(*x*) + *h*(*y*)) holds.
